# Beyond Costs: Pragmatic Considerations for Pooling of SARS-CoV-2 Test Samples for Nursing Home Surveillance

**DOI:** 10.1128/spectrum.03880-22

**Published:** 2023-02-01

**Authors:** Gabrielle M. Gussin, Raveena D. Singh, Delia F. Tifrea, Thomas Tjoa, Cassiana E. Bittencourt, Robert A. Edwards, Edwin S. Monuki, Susan S. Huang

**Affiliations:** a Division of Infectious Diseases, Department of Medicine, University of California Irvine School of Medicine, Irvine, California, USA; b University of California Irvine Health, Orange, California, USA; c Department of Pathology & Laboratory Medicine, University of California Irvine School of Medicine, Irvine, California, USA; University of Mississippi Medical Center

**Keywords:** COVID-19, nursing homes, diagnostics

## Abstract

Pooling of samples can increase throughput and reduce costs for large-scale SARS-CoV-2 testing when incidence is low. In a cross-sectional study of serial SARS-CoV-2 sampling of staff and residents at three nursing homes, laboratory labor constraints limited the feasibility of pooling prior to the maximal incidence that favored cost savings.

**IMPORTANCE** This study highlights the pragmatic considerations surrounding SARS-CoV-2 sample pooling beyond accuracy and costs. We performed a cost analysis to determine the percent positivity at which pooling would reduce costs versus single testing. We found that the need for a stable amount of daily work hours staffed by a highly trained workforce was a major limitation in pooling as test positivity increased. For the COVID-19 pandemic and future pandemic threats, laboratories should carefully consider the thresholds at which sample pooling is beneficial, with a particular focus on the impact on laboratory staff.

## OBSERVATION

Nursing homes (NHs) need efficient and cost-effective methods to identify COVID-19 cases among residents and staff (1, 2). Sample pooling can increase throughput and reduce costs for large-scale SARS-CoV-2 testing (3, 4). We examined the value of pooling for NH COVID-19 surveillance, comparing costs and feasibility of pooling versus individual sample testing at various test positivity levels.

### Pooling sensitivity.

Fifteen of 645 samples (2.33%) were positive for SARS-CoV-2 via initial individual sample testing (“single testing”). The mean cycle threshold (*C_T_*) value across positive samples was 19.97 (standard deviation [SD], 8.02). Banked samples from single testing were randomized into 82 8:1 and 130 5:1 pools. Based upon gold-standard single testing, 17.07% (14/82) of 8:1 pools and 11.54% (15/130) of 5:1 pools had positive samples. Sensitivity and specificity of pooled testing were >90% (Table 1).

**TABLE 1:** Sensitivity and specificity of 8:1 and 5:1 pooling at 2.3% positivity

Pooling characteristics and results	Data for:	
	8:1 pool	5:1 pool
No. of pools	82	130
No. of positive pools		
Actual true positive	14	15
Observed	17	16
No. of false-positive pools	4	2
No. of false-negative pools	1	1
Sensitivity (%)	93.33	93.75
Specificity (%)	94.44	98.29
No. of tests needed to detect 15 of 645 positive samples^*a*^	219	266

a This analysis was based upon 645 anterior nares swabs collected from staff and residents across 3 nursing homes. Of the 645 swabs, 15 (2.3%) were SARS-CoV-2 positive upon initial individual PCR testing.

The single positive sample that failed to be confirmed by either 8:1 or 5:1 pooling had an initial cycle threshold value of 35.90. Screening 645 samples to detect 15 positive samples required 219 tests with 8:1 pooling and 266 tests with 5:1 pooling.

### Pooling costs and feasibility.

**FIG 1:**
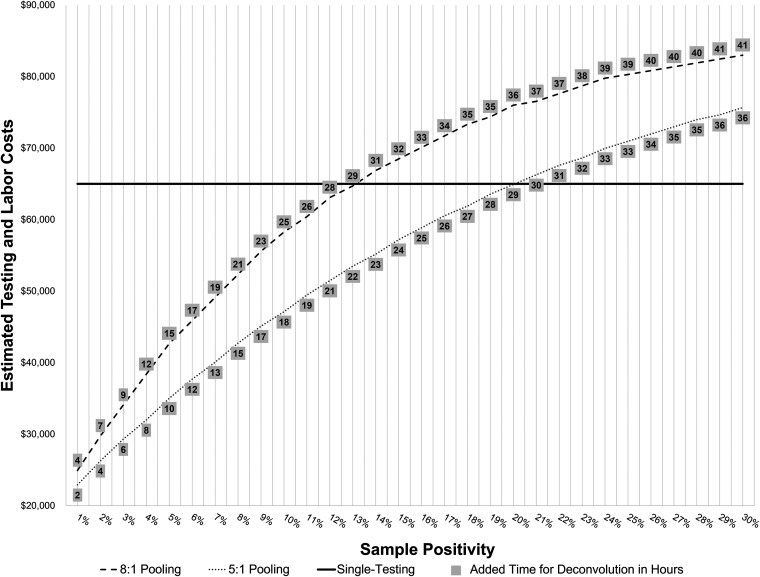
Costs and added person-time associated with 8:1 pooling and 5:1 pooling of 1,000 test samples for SARS-CoV-2 RT-PCR testing and various percent sample positivity. The threshold at which pooling would be cost more than single testing was 14% for 8:1 pooling and 21% for 5:1 pooling. Dashed lines show testing and labor costs for pooling. Solid line shows testing and labor costs for single testing. Gray boxes show the added time in hours for deconvolution of positive pools.

With assurance of high sensitivity and specificity at 2.33% positivity, we estimated costs and person-hours for 8:1 and 5:1 pooling of 1,000 samples across a range of sample positivity (Fig. 1). Compared to the cost of 1,000 single tests ($65,000), the theoretical percent positivity threshold at which the cost of pooling exceeded single testing was 14% for 8:1 pooling and 21% for 5:1 pooling. However, at these theoretical thresholds, the additional person-time needed to deconvolute positive pools was 31 h for 8:1 pooling and 30 h for 5:1 pooling (see numbers in gray boxes in Fig. 1). Despite this theoretical cost savings, clinical laboratory leadership confirmed that at higher percent positivity, the amplitude of uncertainty in positive pools, combined with the cost and logistical challenges of having large numbers of “standby” laboratory staff at all hours to address deconvolution in a timely manner, precluded the use of a pooled sampling strategy above 5% percent positivity.

The results of our study are consistent with prior findings that pooling is efficient when COVID-19 incidence is low (3, 5, 6). We show high sensitivity and specificity with 5:1 and 8:1 pooling, with false-negative pools associated with high PCR cycle thresholds (>35), consistent with very low viral loads. Evidence that these high PCR cycle thresholds often indicate nonviable virus further supports the sufficient sensitivity of pooling with current high-performance PCR platforms (7).

However, we found that the value of pooling was limited when percent sample positivity rose above 5%, even though theoretical cost savings were retained at positivity levels of 14 to 21%. The reasons for this discrepancy are related to person-time requirements that highlight a major pragmatic limitation of pooling. First, it was not feasible to retain laboratory staff “at the ready” at all hours to deconvolute positive pools whenever they arose. The added “hands-on” time needed to locate individual samples when a positive pool needed to be deconvoluted was prohibitive for a high-volume clinical laboratory when COVID-19 incidence was high. This explains why pooling is not commonly used beyond a 1 to 3% incidence (6). Higher uncertainty requires the ability to tolerate sudden changes in staffing needs, scheduling, and provision of overtime pay based upon the daily number of positive pools. A high community COVID-19 incidence also affects staff illness and callouts from work. Furthermore, uncertainty in the daily amplitude of work can produce a psychological toll due to the pressure to maintain or mitigate delays in turnaround time, especially since high percent positivity could be associated with NH outbreaks with meaningful harm if results are not rapidly received to activate infection prevention measures. Thus, the need for a stable amount of daily work hours staffed by a stable and reliable workforce was a major constraint on the upper limit of the daily number of positive pools. If this uncertainty could be managed by 24/7 automation, then cost savings could be achieved at a much higher percent positivity.

The evaluation of samples in a single center limits generalizability. In addition, the use of stored samples may have affected pooling results, although studies have validated the accuracy of PCR testing in frozen SARS-CoV-2 samples (8). Moreover, while we included real-world estimates for costs and person-time requirements, we did not account for issues requiring repeat testing or overtime costs.

Pooling can be a valuable strategy to reduce costs for high-throughput SARS-CoV-2 testing. When test positivity rates are low, pooling offers a cost-efficient and accurate way to detect COVID-19 cases among NH residents and staff. Importantly, this study highlights the pragmatic considerations surrounding sample pooling beyond accuracy and costs. For the COVID-19 pandemic and future pandemic threats, laboratories should carefully consider the thresholds at which sample pooling is beneficial, with a particular focus on the impact on laboratory staff.

### Pooling sensitivity and specificity.

We conducted a cross-sectional study of routine weekly COVID-19 testing sweeps of residents and staff in a convenience sample of three NHs in Orange County, California, to evaluate the sensitivity of sample pooling for NH surveillance. Sweeps were conducted from 6 January 2021 to 21 January 2021 as a nonresearch public health endeavor within our role as the county’s NH COVID-19 Prevention Team (9). Testing involved bilateral anterior nares swabs collected in Abbott Universal multi-Collect sample collection kits and processed individually for real-time reverse transcriptase PCR (RT-PCR) testing of SARS-CoV-2 open reading frame 1 (ORF1), N, and S genes by the University of California Irvine Medical Center (UCIMC) clinical molecular laboratory. After processing, all swabs (*n* = 645) were stored at −80°C for pooling analysis. Results from individually processed (single-tested) swabs informed the value of pooling studies from banked samples.

Within NH strata, the 645 stored samples were randomly assigned into 8:1 and 5:1 pools and tested. These 645 samples represent all staff and residents tested across the three NHs included in this analysis. Positive pools were deconvoluted into individual RT-PCR runs. All testing was performed using the TaqPath COVID-19 Combo kit (Thermo Fisher, Waltham, MA) on the Applied Biosystems 7500 Fast DX real-time PCR instrument (limit of detection, 3; *C_T_*, 250 copies/mL; sensitivity, 95%). False-positive and false-negative pools were determined using single-test results as the gold standard, and the sensitivity and specificity of pooling were calculated. Cycle threshold values from single-testing were available for comparison purposes.

### Estimated cost savings by pooling by percent positivity of samples.

We performed a cost analysis to determine the percent positivity at which pooling would reduce costs versus single testing. UCIMC clinical laboratory costs and person-hours required for 8:1 and 5:1 sample pooling of 1,000 samples were compared to single testing, with test positivity ranging from 1 to 30%. In this exercise, each assigned percent positivity was translated to positive pools per 1,000 samples and randomly distributed across pools in 500 simulated iterations. For each pool, we evaluated the following: [cost to set up pools ($0.55/sample)] + [total pools × testing cost ($19/pool)] +{labor cost to deconvolute positive pools into single-test runs[2.20/sample (2.6 min/sample) for locating and priming positive samples for testing]} + [estimated number of positive pools × total samples per pool × single-test cost ($65/sample)]. The cost of $65/sample per test comes from actual labor and material costs from UCIMC. Using these values, we identified the positivity threshold at which the cost of pooling exceeded single testing.
